# Crystal Structures of the Sec1/Munc18 (SM) Protein Vps33, Alone and Bound to the Homotypic Fusion and Vacuolar Protein Sorting (HOPS) Subunit Vps16*

**DOI:** 10.1371/journal.pone.0067409

**Published:** 2013-06-26

**Authors:** Richard W. Baker, Philip D. Jeffrey, Frederick M. Hughson

**Affiliations:** Department of Molecular Biology, Princeton University, Princeton, New Jersey, United States of America; Institute of Molecular and Cell Biology, Biopolis, United States of America

## Abstract

Intracellular membrane fusion requires the regulated assembly of SNARE (soluble N-ethylmaleimide-sensitive factor (NSF) attachment protein receptor) proteins anchored in the apposed membranes. To exert the force required to drive fusion between lipid bilayers, juxtamembrane SNARE motifs zipper into four-helix bundles. Importantly, SNARE function is regulated by additional factors, none more extensively studied than the SM (Sec1/Munc18-like) proteins. SM proteins interact with both individual SNAREs and SNARE complexes, likely chaperoning SNARE complex formation and protecting assembly intermediates from premature disassembly by NSF. Four families of SM proteins have been identified, and representative members of two of these families (Sec1/Munc18 and Sly1) have been structurally characterized. We report here the 2.6 Å resolution crystal structure of an SM protein from the third family, Vps33. Although Vps33 shares with the first two families the same basic three-domain architecture, domain 1 is displaced by 15 Å, accompanied by a 40° rotation. A unique feature of the Vps33 family of SM proteins is that its members function as stable subunits within a multi-subunit tethering complex called HOPS (homotypic fusion and vacuolar protein sorting). Integration into the HOPS complex depends on the interaction between Vps33 and a second HOPS subunit, Vps16. The crystal structure of Vps33 bound to a C-terminal portion of Vps16, also at 2.6 Å resolution, reveals the structural basis for this interaction. Despite the extensive interface between the two HOPS subunits, the conformation of Vps33 is only subtly affected by binding to Vps16.

## Introduction

Vesicular transport and homotypic fusion depend on the docking and fusion of membranes, processes that are mediated by SNARE proteins working in conjunction with a host of regulatory factors [1], [2]. Prominent among these regulatory factors are the 60- to 70-kDa Sec1/Munc18 (SM) proteins [3], [4]. One of the first SM proteins, Sec1, was discovered in a screen for yeast secretory pathway components [5], [6]; subsequent work has implicated SM proteins in the assembly and/or function of many if not all SNARE complexes. There are four families of SM proteins – Sec1/Munc18, Sly1, Vps33 and Vps45– thought to operate in conjunction with distinct sets of SNAREs. At least one SM protein from each family is present in most eukaryotes [7], [8]. A number of crystal structures have been reported for SM proteins of the Sec1/Munc18 and Sly1 families [9]–[16]. These structures reveal three domains, arranged in an arch-like configuration, surrounding a central cleft implicated in SNARE binding.

SNARE proteins contain a 60- to 70-residue SNARE motif, almost always located directly adjacent to a transmembrane anchor [4], [17]. SNARE motifs from different SNARE proteins assemble to form four-helix bundles that bridge membranes and mediate their fusion. The core of the SNARE four-helix bundle consists almost exclusively of non-polar amino acids, except at the central ‘zero’ layer where the four core residues are either glutamine (Q) or arginine (R). Depending on the identity of the zero-layer residue and its position within the bundle, SNAREs are classified as Qa- (or syntaxin-like), Qb-, Qc-, or R-SNAREs.

The first reported SM protein structure contained both Munc18–1 (also called Munc18a or neuronal Sec1) and the Qa-SNARE syntaxin 1A [16]. Syntaxin 1A bound to Munc18–1 adopts a closed conformation, with a portion of its SNARE motif and its N-terminal regulatory domain – a three-helix bundle – combining to form a four-helix bundle. The four-helix bundle of closed syntaxin 1A nestles within the Munc18–1 cleft. Several studies suggest that the SM cleft binds not only the four-helix bundles formed by closed Qa-SNAREs but also the four-helix bundles formed by fully assembled SNARE complexes [18]–[27]. No direct structural evidence supporting this suggestion has, however, been reported. A second major mode of SM:SNARE interaction entails the binding of some, but not all, SM proteins to a conserved peptide motif located near the N-terminus of the Qa-SNARE [11]–[15], [28]–[30]. The binding site for the N-peptide is a groove on the surface of domain 1 that is remote from the cleft. In at least some cases, Qa-SNAREs bind to SM proteins using both modes simultaneously, with the closed four-helix bundle in the cleft and the extended N-terminus reaching around domain 1 to bind in the distal groove [12], [13].

There is strong evidence that SM proteins do not function alone. Recent biochemical reconstitutions imply that Munc18–1 works together with Munc13 to chaperone the assembly of neuronal SNARE complexes [31], [32]. Genetic and biochemical interactions connect the SM protein Sly1 with two large multisubunit tethering complexes, the COG (conserved oligomeric Golgi) and Dsl1 complexes [33], [34]. Interestingly, both COG and Dsl1 complexes contain multiple subunits that are structurally homologous to Munc13 [35]. Despite mounting evidence for collaboration, however, only one SM protein – Vps33– is known to form a stable complex with other polypeptides [36]. Vps33 regulates fusion in the endo-lysosomal membrane system as a stable subunit within two large multisubunit tethering complexes, HOPS (homotypic fusion and vacuolar protein sorting) and CORVET (class C core vacuole/endosome tethering) [36]–[40]. HOPS has been intensively studied; notably, it is required for SNARE-mediated membrane fusion in a biochemically reconstituted system [41].

Recently, the overall structure of the HOPS complex at a resolution of approximately 29 Å was determined using electron microscopy combined with single-particle analysis and tomography [42]. As a next step toward a better mechanistic understanding of the role of Vps33 in HOPS/CORVET function, we here report the crystal structure of Vps33 from the thermophilic fungus *Chaetomium thermophilum*. To elucidate the structural basis for the integration of Vps33 into the HOPS/CORVET complexes, we also determined the crystal structure of Vps33 bound to a C-terminal domain of *C. thermophilum* Vps16.

## Materials and Methods

### Protein Production and Purification

*Chaetomium thermophilum* Vps16 (NCBI accession EGS20838) and Vps33 (NCBI accession EGS19151) were identified by homology with *Saccharomyces cerevisiae* Vps16 and Vps33 using the *C. thermophilum* genome resource website (http://ct.bork.embl.de/). Coding sequences were amplified from *C. thermophilum* cDNA (a generous gift of Dr. Ed Hurt) and cloned into the pQLinkH and pQLinkN bacterial expression plasmids (Addgene plasmids 13667 and 13670, respectively) [43]. The resulting pQLinkH plasmids encode fusion proteins with N-terminal heptahistidine tags and tobacco etch virus (TEV) protease cleavage sites for tag removal, whereas the corresponding pQLinkN plasmids encode untagged proteins. A plasmid for the co-expression of His7-Vps33 and Vps16_CTD_ (residues 505–834) was created by sub-cloning the appropriate region of the pQLinkH-Vps16 plasmid into pQLinkN and using the pQLink combination protocol [43]. Vps33 mutants were generated by site-directed mutagenesis [44].

Native and selenomethionine- (SeMet-) substituted proteins were over-produced in BL21 Rosetta bacteria (Novagen) in, respectively, LB or M9 minimal media supplemented with 60 mg/liter SeMet (Sigma). Cells were grown at 37°C until the OD_600_ reached approximately 0.6 and then induced with 0.5 mM IPTG at 25°C for 18 h. Cell pellets were resuspended in lysis buffer (50 mM Tris-HCl, pH 7.4, 250 mM NaCl, 20 mM imidazole, 1 mM dithiothreitol (DTT)) supplemented with 1 mM phenylmethylsulfonyl fluoride, 10 µg/ml DNase (Roche), and 1 mg/ml lysozyme (Sigma). After 30 min at 24°C, the resuspension was processed with an Emulsiflex-C5 homogenizer (Avestin). All subsequent steps were performed on ice or at 4°C. The cell lysate was clarified by centrifugation at 30,000 g and fractionated using His60 Ni Superflow Resin (ClonTech). His_7_-fusion proteins were eluted using lysis buffer with the addition of 400 mM imidazole and cleaved overnight using recombinant His_7_-TEV protease at a 1∶50 (w/w) ratio. After dialysis to reduce the salt concentration to 100 mM, another round of Ni^2+^ affinity chromatography was used to remove the protease and any uncleaved His_7_-fusion protein. The resulting untagged proteins were further purified using SourceQ 10/10 anion exchange and Superdex 200 HR 10/30 size exclusion columns (GE Healthcare). On the latter column, all proteins eluted as single symmetric peaks at volumes indicative of monomers (Vps33, Vps16) or heterodimers (Vps16_CTD_–Vps33). After concentration, proteins stocks (approximately 5 mg/ml protein in 20 mM Tris-HCl, pH 7.4, 250 mM NaCl, 1 mM DTT) were stored at −80°.

### Crystallization and Structure Determination

Vps33 crystals were grown at 20°C using the sitting drop vapor diffusion method with a 1∶1 (v/v) mixture of protein (5 mg/ml) and precipitant solution (0.2 M potassium citrate, 12–18% (w/v) PEG 3350, 10 mM barium chloride). Both native and SeMet protein crystals grew under the same conditions. Crystals were improved by streak seeding with native crystals and grew to full size in approximately 72 h. Unit cell dimensions were a = 71.9 Å, b = 64.4 Å, c = 151.7 Å, β = 91.8° in space group P2_1_, with two molecules in the asymmetric unit. Vps16_CTD_–Vps33 crystals were grown at 20°C using the sitting drop vapor diffusion method with a 1∶1 (v/v) mixture of protein (5 mg/ml) and precipitant solution (0.1 M MES, pH 6.0, 150 mM ammonium sulfate, 12–16% (w/v) PEG 4000). Crystals were improved by streak seeding and grew to full size in 18 h. Unit cell dimensions were a = b = 100.3 Å, c = 176.2 Å in space group P3_2_21, with a single complex in the asymmetric unit. SeMet anomalous diffraction data were collected at the inflection and high energy remote wavelengths of the Se K edge using beamline X29 of the National Synchrotron Light Source at Brookhaven National Laboratory. Data for Vps16_CTD_–Vps33 were processed using the HKL suite [45]; data for Vps33 were processed using autoPROC [46], employing XDS [47] for data integration and SCALA [48] for scaling (Table 1).

**Table 1:** Crystallographic data, phasing and refinement statistics.

Data Collection	Vps33 SeMet MAD^a^		Vps16 SeMet
Data set	Se Inflection	Se Remote	Se Inflection
Wavelength (Å)	0.9793	0.9640	0.9793
Resolution (Å)	50.−2.66 (2.74–2.66)^b^	50.−2.60 (2.74–2.60)^b^	50.−2.60(2.64–2.60)^b^
Unique reflections	40,688	43,028	31,686
Completeness (%)	99.8 (99.9)^b^	99.8 (99.9)^b^	98.1 (94.0)^b^
Redundancy	3.7 (3.8)^b^	3.7 (3.7)^b^	9.3 (9.4)^b^
R_sym_ (%)			11.1 (87.3)^b^
R_meas_ (%)	8.5 (63.3)^b^	9.0 (61.1)^b^	
<I/σ_I_>	16.1 (2.5)^b^	15.1 (2.6)^b^	13.7 (2.4)^b^
**Phasing**			
Figure of Merit	0.52		N/A
**Refinement**			
Resolution		50.−2.60 (2.66–2.60)^b^	50–2.60(2.68–2.60)
Number of reflections(free set)		43,008 (2,153)	31,605(1,600)
Completeness (%)		99.7	98.1
R-factor (%)		18.5 (24.9)^b^	22.6 (26.3)^b^
R-free (%)		24.7 (33.3)^b^	25.4 (33.1)^b^
Number of			
Protein atoms		9,565	6,658
Water atoms		225	147
RMSD_bond_ (Å)^c^		0.009	0.002
RMSD_angle_ (°)^c^		1.218	0.634
Wilson B factor (Å^2^)		46.7	56.8
Average B factor (Å^2^) of			
All atoms		25.2	61.0
Main chain		24.7	60.9
Side chain		26.3	61.1
Waters/Sulfate		19.2	59.4
Ramachandran plot, residues in (%)			
Favored		95.2	93.2
Allowed		3.8	5.1
Outliers		1.0	1.7
PDB entry^d^		4JC8	4KMO

a Multiwavelength anomalous diffraction.

b Values in parentheses are for the highest resolution shell.

c RMSD, root mean square deviation.

d Protein Data Bank.

For Vps33, the positions of thirteen Se atoms were determined using the program SHELXD [49] and phases were improved using the program SHARP [50]. The structure of Vps33 was built into experimentally-phased maps using the program COOT [51] and refined with PHENIX [52] using non-crystallographic symmetry restraints between the two molecules in the asymmetric unit (Table 1). Both experimentally-phased and model-phased maps were averaged using the Uppsala Software Factory suite [53]; the program LSQMAN was used for structure superimpositions [54]. The current model contains coordinates for residues 5–654 in each of the two independent molecules (chains A and B). The following residues are omitted from the model as no interpretable electron density was present: A1–4, A213–222, A277–295, A339–343, A547–555, A583–599, A655–667, B1–4, B213–222, B277–295, B543–558, B584–599, and B655–667.

The Vps16_CTD_–Vps33 structure was determined by the method of molecular replacement using the program PHASER [55] and, as a search model, the Vps33 monomer. Vps16 was built into model-phased 2Fo-Fc and Fo-Fc electron density maps. Sequence assignment was guided by Se locations obtained from SeMet SAD data (Table 1). The quality of the electron density maps calculated from SAD- and MAD-derived experimental phases were inferior to the model-phased maps, but confirmed the topology of Vps16_CTD_. The Vps16_CTD_–Vps33 structure was built and refined using COOT and PHENIX, respectively. The current model contains coordinates for Vps16_CTD_ residues 520–791 and Vps33 residues 5–657 with one sulfate anion and 147 waters (Table 1). The following residues are omitted from the model as no interpretable electron density was present: Vps16–505–519, 598–604, and 792–834; Vps33–1–4, 213–217, 271–295, 334–356, 547–555, 583–599, and 658–667.

### Binding Experiments

Binding between full-length Vps33 (wild-type or mutant) and full-length Vps16 was evaluated using size exclusion chromatography. Approximately 75 µM Vps33 and Vps16 were incubated together at 25°C for 1 h before injecting onto a Superdex 200 HR 10/30 column.

### Modeling a SNARE Complex into the Major Groove of Vps33

We constructed a hypothetical model of a Vps33–SNARE complex based on two published structures: the *Monosiga brevicollis* Munc18–syntaxin 1 complex (PDB entry 2XHE) [13] and the neuronal synaptic SNARE complex (PDB entry 1SFC) [56]. In each of these structures, the SNARE or SNARE complex forms a four-helix bundle. The topologies of these bundles are, however, different: in closed syntaxin 1, the helices alternate in orientation, whereas in the SNARE complex all four helices are parallel. Furthermore, while the syntaxin 1 SNARE motif is present in both structures, its conformation is substantially different. We therefore docked the SNARE complex onto the Munc18–syntaxin 1 structure by manually optimizing the overlap between the helical bundles. Our model aligns the following helical regions: residues 163–181 of SNAP-25B with residues 50–68 of syntaxin 1; residues 48–79 of synaptobrevin 2 with residues 89–120 of syntaxin 1; residues 32–73 of SNAP-25B with residues 130–173 of syntaxin 1; and residues 218–233 of syntaxin 1A with residues 221–236 of syntaxin 1. Vps33 was positioned by aligning domains 2 and 3 with the corresponding domains of Munc18. We also modeled a closed conformation of Vps33 by replacing residues 332–356 with the corresponding region (residues 304–337) of Munc18. We emphasize that the resulting Vps33–SNARE complex models are not sufficiently well constrained to use as bases for predicting the detailed interactions between the SNARE complex and Vps33. Instead, they are intended to illustrate the general features that such a complex would possess, assuming that a SNARE bundle bound to Vps33 were to occupy the same general position as the syntaxin 1 helix bundle bound to Munc18.

## Results

We began our structural studies of the HOPS complex with two of its six subunits, Vps16 and the SM protein Vps33. Vps16 and Vps33 form a stable sub-complex [42], [57], [58] and represent two of the three subunits (the third being Vps18) that have been implicated in interactions with vacuolar SNAREs [21], [22], [59], [60]. To determine the structures of Vps33 and Vps16–Vps33, we adopted an approach with a long history in studies of bacterial proteins but that has only recently been applied to eukaryotic proteins – the use of orthologs derived from thermophilic organisms [61], [62]. We found that Vps16 and Vps33 from *Chaetomium thermophilum*, overproduced in *E. coli*, were highly soluble and monodisperse; combined, they formed Vps16–Vps33 complexes that were likewise monodisperse (see Materials and Methods).

### Structure of *C. thermophilum* Vps33

**Figure 1:**
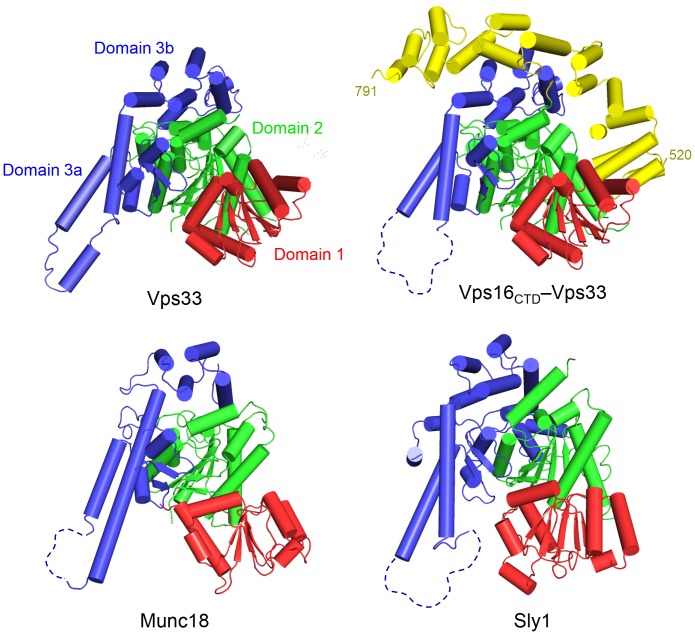
SM protein family comparison. Vps33, Vps16_CTD_–Vps33, rat Munc18–1 (PDB entry 3PUJ), and Sly1 (1MQS) are shown with cylinders representing α-helices. The different position of domain 1 in Vps33, relative to Munc18–1 and Sly1, is especially notable. The tip of domain 3a shows varying degrees of disorder, as discussed in the text.

**Figure 2:**
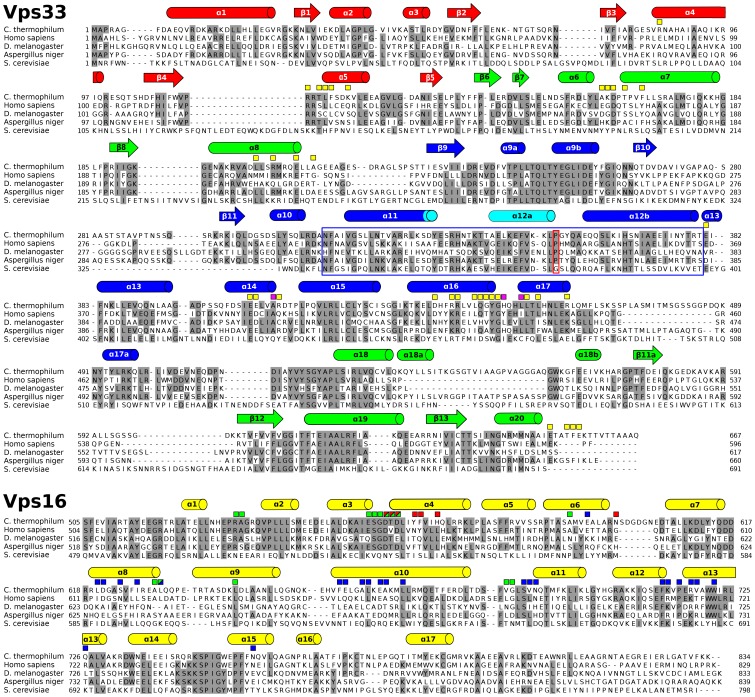
Sequence alignment for Vps33 and the C-terminal region of Vps16. Intermolecular contacts (<4 Å) are indicated using boxes. For Vps33, these boxes are yellow except for those residues depicted in magenta in Fig. 3B. For Vps16, boxes are color coded to match the Vps33 domain(s) contacted by a given residue. The distal tip of Vps33 domain 3a is highlighted with a blue box. The ‘hinge’ proline (see Fig. 6 legend) is highlighted with a red box. Secondary structural assignments for Vps33 are based on [16] and are colored by domain as in Fig. 1; helix α12 is shown in light blue to indicate that it is ordered in Vps33 but not in Vps16_CTD_–Vps33 (see text for details). Sequence alignments were performed using CLUSTALW [68] on 15 Vps33 and 15 Vps16 orthologs; for clarity, only 5 orthologs are shown here. The orthologs shown (with percentage sequence identity for Vps33/Vps16 listed in parentheses) are: *Homo sapiens* (37/33), *Drosophila melanogaster* (30/27), *Aspergillus niger* (61/58), and *Saccharomyces cerevisiae* (19/20).

The structure of *C. thermophilum* Vps33 was determined using MAD phasing and refined to 2.6 Å resolution (Table 1; see Materials and Methods). Vps33 shares the overall shape and topology observed for previously reported SM protein structures (Fig. 1) [9]–[16]. Like these other SM proteins, Vps33 is arranged in an arch-shaped configuration with overall dimensions of 60 x 65 x 80 Å. Following the nomenclature introduced by Misura et al. [16], Vps33 contains three domains (Figs. 1 and 2): domain 1 (residues 1–138; red), domain 2 (residues 139–248 and 502–667; green), and domain 3 (residues 249–501; blue), with domain 3 further subdivided into 3a (residues 249–380) and 3b (residues 381–501). The two copies of Vps33 present within each asymmetric unit of the crystal are highly similar to one another and can be superimposed with a root-mean-squared deviation of only 0.56 Å (over 580 Cα atoms). Despite this very high degree of overall similarity, a functionally important region of domain 3a adopts somewhat different conformations in the two Vps33 monomers, as discussed below.

### Structure of *C. thermophilum* Vps16_CTD_–Vps33

Like all of the HOPS/CORVET subunits except Vps33, Vps16 is predicted to contain an N-terminal β-propeller followed by an α-solenoid. Many of the HOPS/CORVET subunits, but not Vps16, also contain a RING or RING-like motif near their C-termini [40], [58]. We were unsuccessful in generating useful crystals of full-length *C. thermophilum* Vps16, either alone or in complex with Vps33. As an alternative, we co-expressed and crystallized Vps33 with a C-terminal fragment of Vps16 (Vps16_CTD_; residues 505–834). The corresponding fragment of the *S. cerevisiae* ortholog, containing ∼60% of the predicted α-solenoid domain, was shown previously to bind Vps33 [58]. The structure of the *C. thermophilum* Vps16_CTD_–Vps33 complex was determined by molecular replacement using *C. thermophilum* Vps33 as a search model and was refined to 2.6 Å resolution (Table 1; see Materials and Methods).

Vps16_CTD_ contains an irregular α-solenoid made up of 17 α-helices (Figs. 1 and 2) arranged in a manner similar to HEAT repeat proteins. Following helix α5, there is an abrupt change in helix orientation, such that the Vps16_CTD_ structure can be viewed as containing two distinct regions, α1-α5 and α6-α17. An additional irregularity is evident after the unusually long α10 helix: the following antiparallel helix is absent, replaced instead by a region of extended structure. A survey of the Protein Data Bank using the Dali server [63] revealed that Vps16_CTD_ displays weak structural homology to other HEAT-repeat-like structures, including a nuclear pore subunit (Nup120; Z = 8.0) and vesicle coat proteins (clathrin heavy chain and α-COP; Z = 6.9 and 6.3, respectively; Fig. S1).

**Figure 3:**
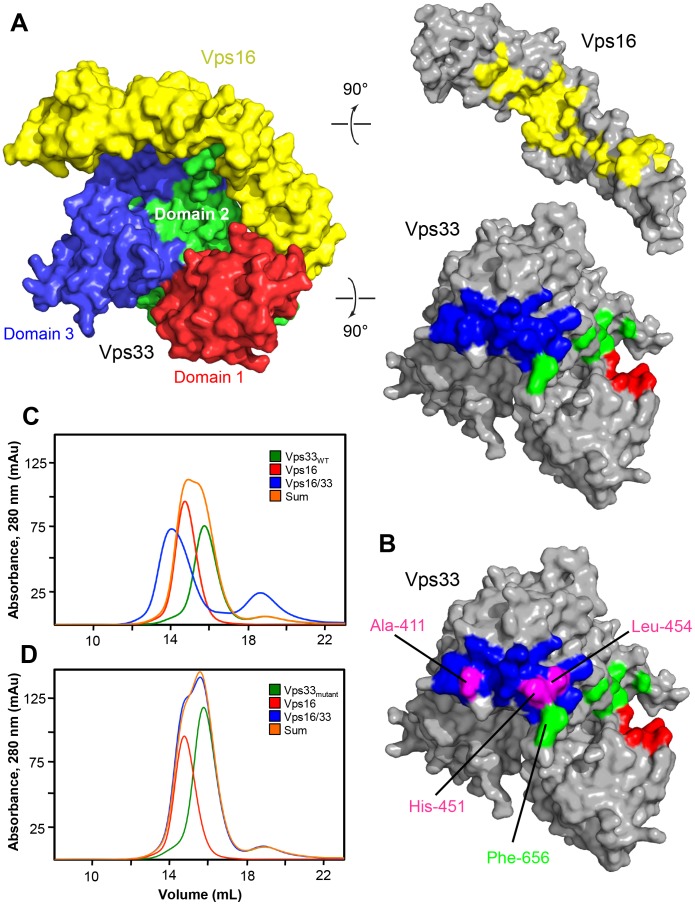
Interaction between Vps16_CTD_ and Vps33. (A) Vps16_CTD_ and Vps33, oriented as in Fig. 1, are separated and rotated to reveal the contact surfaces. (B) In magenta are shown the positions of Vps33 residue substitutions engineered to disrupt the complex. Also indicated is Phe-656, one of three residues near the C-terminus of Vps33 (and therefore located in domain 2) that is well-ordered only in the Vps16_CTD_–Vps33 complex. (C) Size exclusion chromatography was used to analyze wild-type Vps33, full-length Vps16, and the combination of the two. Shown for comparison is the sum of the chromatograms for the individual proteins. The Vps16–Vps33 complex elutes earlier from the column, consistent with its larger size. (D) As in panel C, but with Vps33 A411D/H451D in place of wild-type Vps33. The binding reaction is indistinguishable from the sum of the individual protein chromatograms, indicating the absence of a detectable interaction. The same result was obtained for Vps33 A411D/L454E (not shown).

Vps16_CTD_ binds to the upper surface of the Vps33 arch, opposite the large cleft between Vps33 domains 1 and 3 (Fig. 1). In so doing, it interacts with all three domains of Vps33, burying about 4800 Å^2^ of accessible surface area in the interface (Fig. 3A). The N-terminal portion of Vps16_CTD_ lies in the groove between domains 1 and 2 of Vps33; the majority of the Vps16_CTD_–Vps33 contacts in this region involve polar rather than hydrophobic residues. Almost all of the contacts with domain 1 are made by Vps16 helix α4 (Fig. 2). By contrast, helices α1-α4 approach domain 2 end-on, such that the majority of the contacts are made by the α1-α2 and α3-α4 loops.

The contact surface with domain 3b entails a mixture of polar and non-polar interactions involving Vps16 helices α8-α13; the long α10 helix packs especially extensively against Vps33 (Fig. 2). Interestingly, three residues near the C-terminus of Vps33 (residues 655–657), which are disordered in the uncomplexed protein, adopt a well-ordered conformation in the Vps16_CTD_–Vps33 complex. One of these, Phe-656 (Fig. 3B), fits into a hydrophobic pocket comprising Vps16 residues Ala-630, Leu-631, Leu-671, and Val-686. The only domain of Vps33 that does not interact directly with Vps16_CTD_ is domain 3a; we return to the significance of this observation below. Nonetheless, despite the extensive interaction between Vps33 and Vps16_CTD_, the backbone conformation of Vps33 is almost identical to that observed for the uncomplexed protein (root-mean-squared deviation <0.6 Å). The most significant concerted shifts are still very small (<1 Å) but involve three α-helices in domain 3b (α14, α16, and α17) that form part of the interface with Vps16.

To validate the crystallographically observed complex, we tested binding of full-length Vps16 to wild-type Vps33 and to Vps33 mutants designed to disrupt Vps16 binding (Fig. 3B). Wild-type Vps33 forms a complex with full-length Vps16 that is readily detected by gel filtration (Fig. 3C). Conversely, whereas three single-residue substitutions in domain 3b – A411D, H451D, and L454E – failed to entirely disrupt complex formation, combining these mutations in pairs yielded Vps33 mutant proteins (A411D/H451D and A411D/L454E) that displayed no detectable complex formation (Fig. 3D and data not shown). The apparent stability and chromatographic behavior of the mutant Vps33 proteins themselves are indistinguishable from wild-type (Fig. 3C,D and data not shown). Taken together, these structural and biochemical data confirm the earlier conclusion, based on yeast two-hybrid experiments [58], that Vps16 residues 505–834 (residues 479–798 in *S. cerevisiae*) are both necessary and sufficient for binding to Vps33.

### Major Repositioning of Domain 1

**Figure 4:**
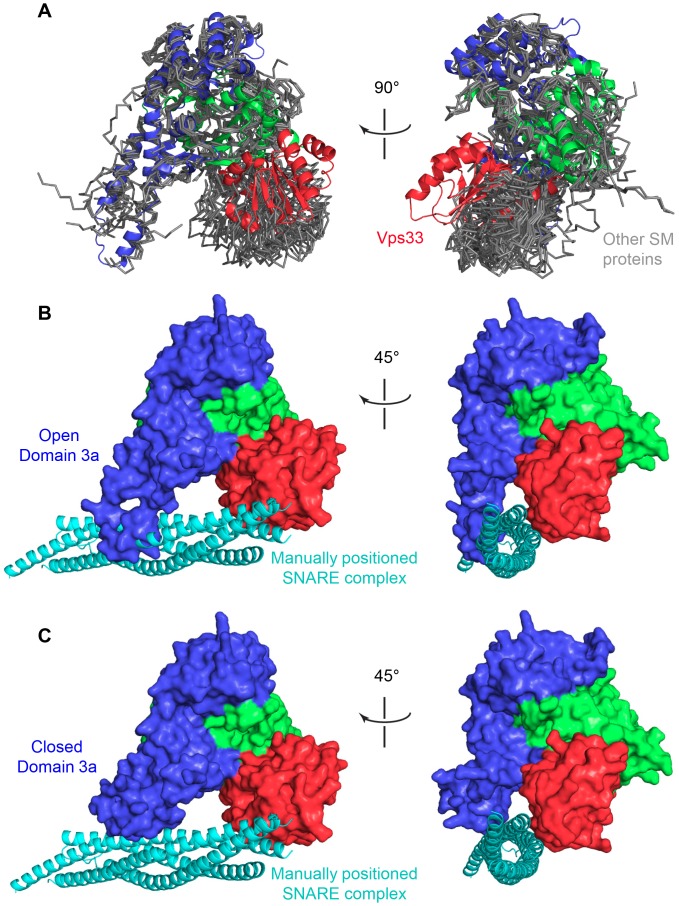
The position of Vps33 domain 1 is unique among known SM proteins. (A) All known SM protein structures (PDB entries 1EPU, 1FVF, 1MQS, 2XHE, 3C98, 3PUJ, and 3PUK), including multiple molecules within the asymmetric unit (whenever present), were aligned with Vps33 based on domains 2 and 3. Vps33 is shown in ribbon representation, colored as in Fig. 1; all other SM proteins are shown in simple representation and colored gray. (B) Using the structure of Munc18–1 in complex with syntaxin 1 (2XHE), a ternary SNARE complex (1SFC) was modeled into the central cleft of Vps33. (C) As in panel B, but with the tip of Vps33 modeled in a closed conformation. For model generation, see Materials and Methods.

The positioning of domain 1 in Vps33 is substantially different from that observed in all previously determined SM protein structures (Fig. 4A). This difference is highly unlikely to reflect inter-domain flexibility, as the position and orientation of domain 1 is virtually identical in the two independent molecules contained within each asymmetric unit of the monomeric Vps33 crystals, as well as in the Vps16_CTD_–Vps33 structure. For example, after domains 2 and 3 of the two independent copies of monomeric Vps33 are superimposed, the two copies of domain 1 differ by only a 2.2° rotation and a 0.6 Å translation. Much larger rotations (36–52°) and translations (12–15 Å) are observed when the same procedure is used to compare Vps33 to the other known SM proteins. These differences are substantially larger than the variation among Munc18 structures that led Bracher and Weissenhorn [10] and Hu et al. [14] to propose a hinge between domains 1 and 2. Thus, in terms of the position and orientation of domain 1, Vps33 is an outlier among known SM protein structures (Fig. 4A). Furthermore, the unprecedented positioning of domain 1 does not depend on the presence (or absence) of Vps16.

We sought to evaluate how the repositioning of domain 1 might affect SNARE binding. Unlike Munc18–1, Vps33 does not bind to closed Qa-SNAREs [59]; instead, recent evidence suggests that it binds to ternary Q-SNARE and quaternary Q/R-SNARE complexes [21], [22]. Since there is no reported structure of an SM protein bound to a SNARE complex, we constructed a simple hypothetical model of such a complex based on the assumption that a SNARE complex would bind in a site and orientation analogous to those observed for the four-helix bundle of the Qa-SNARE syntaxin 1 bound to Munc18 [12], [13], [16]. This modeling exercise (see Materials and Methods for details) revealed no significant clashes between the modeled SNARE complex and the repositioned domain 1 of Vps33 (Fig. 4B). A major clash was observed between the SNARE complex and domain 3 of Vps33 but, as described below, there is a precedent for supposing that this region of Vps33 adopts an alternative, ‘closed’ conformation in order to allow SNARE complex binding (Fig. 4C).

### Structural Basis for the Failure of Vps33 to Bind Qa-SNARE N-peptides

**Figure 5:**
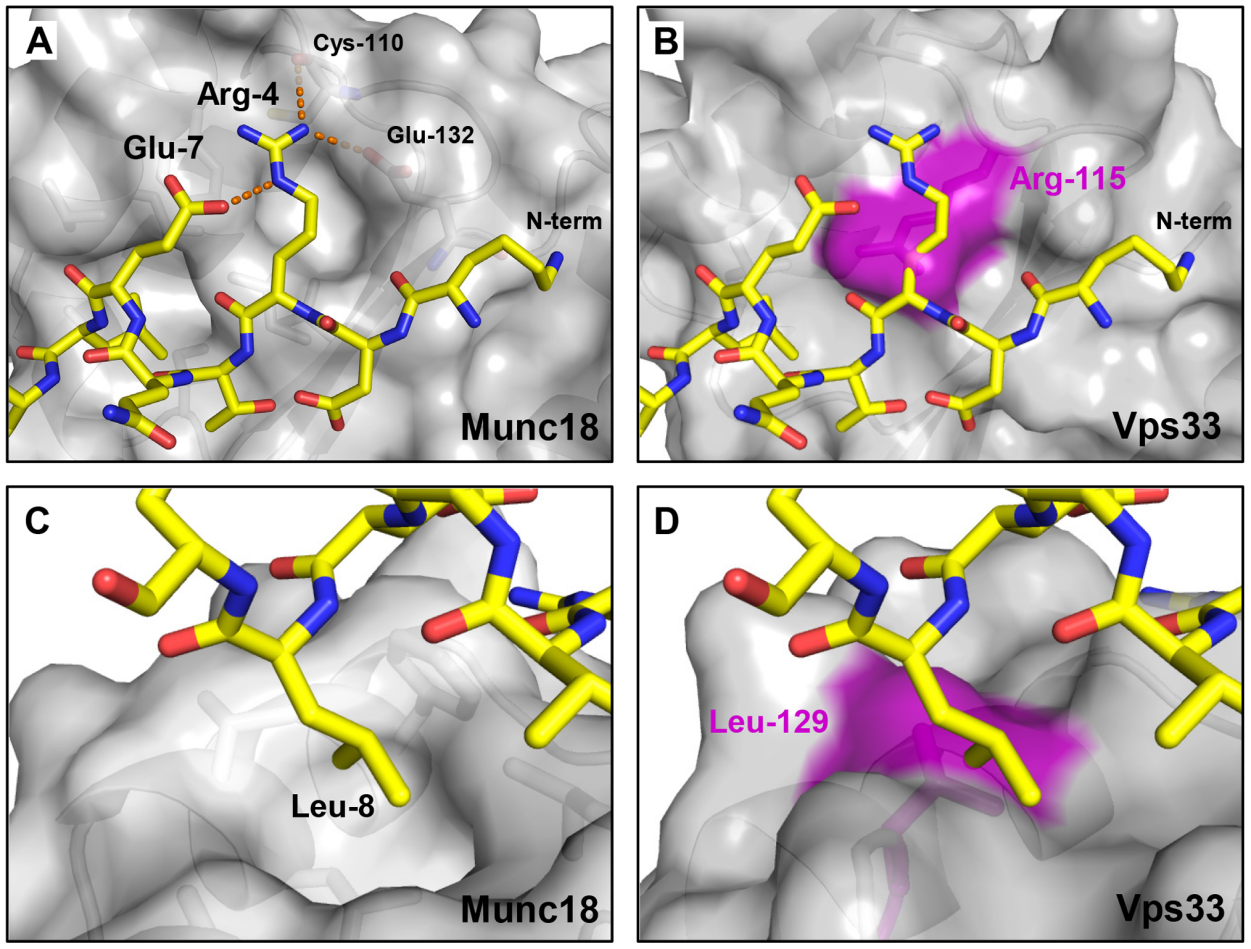
Alterations in Vps33 domain 1 eliminate the N-peptide binding site. (A) Arg-4 plays a key role in the binding of the N-peptide of syntaxin 1A to domain 1 of Munc18–1 (PDB entry 3C98) [12], forming a network of hydrogen bonds and salt bridges denoted by dashed orange lines. (B) The same peptide, overlaid on the corresponding surface of Vps33, clashes with Vps33 residue Arg-115 (purple). (C) A different view of the complex shown in panel A highlights the hydrophobic pocket into which Leu-8 of syntaxin 1A packs. (D) The corresponding view of the model shown in panel B illustrates that Vps33 residue Leu-129 fills the hydrophobic residue binding pocket.

The Vps33 family of SM proteins, unlike the Sec1/Munc18, Sly1, and Vps45 families [11]–[13], [15], [28], [29], does not appear to interact with the N-peptides of Qa-SNAREs [15], [59]. The structural basis for this key distinction among SM protein families can be understood by superimposing domain 1 of Vps33 upon that of other, N-peptide-binding SM proteins. This analysis reveals that the binding site normally occupied by the N-peptide’s conserved Arg side chain (Fig. 5A) is, uniquely in the case of Vps33, filled by Arg-115 of the SM protein itself (Fig. 5B). The positioning of the Arg-115 side chain is reinforced by a salt-bridge formed with Asp-120. Also blocked – and only in Vps33– is the pocket that normally accommodates a conserved hydrophobic residue located four residues C-terminal to the N-peptide Arg residue (Fig. 5C,D). In this case a displacement of the Vps33 backbone, relative to other SM proteins, causes the side chain of Leu-129 to occupy the binding pocket. Thus, both of the pockets that in other SM proteins accommodate conserved Qa-SNARE N-peptide residues are missing in Vps33. Likewise, the N-terminal region of the relevant Qa-SNARE Vam3 lacks the sequence determinants – including the conserved Arg – found in the N-peptides of the Qa-SNAREs that bind SM proteins.

### Domain 3a Contains a Conserved, Yet Flexible, Region Implicated in SNARE Binding

**Figure 6:**
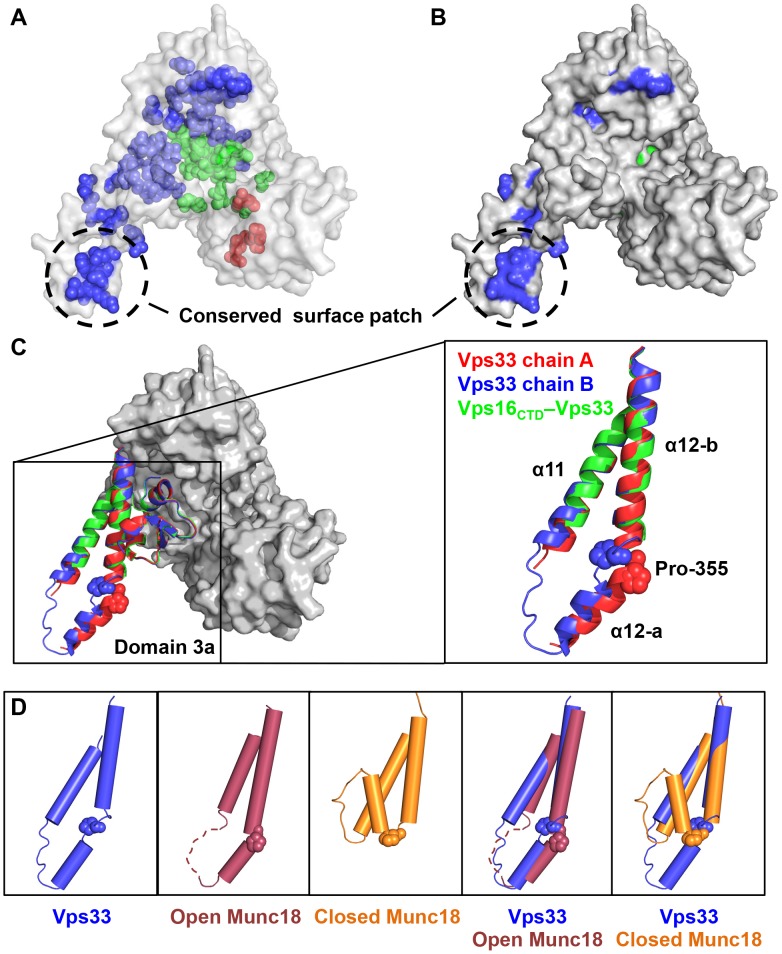
Domain 3a displays an open conformation featuring conserved surface-exposed residues. (A) Highly conserved residues were determined by comparing the sequences of fifteen Vps33 orthologs from yeast to human and are shown on the *C. thermophilum* structure as spheres. (B) A surface representation reveals that a majority of the conserved surface-exposed residues map to domain 3a. Except in domain 3a, few surface-exposed conserved residues are visible on the ‘back’ side of Vps33 (not shown). (C) The two Vps33 monomers present in the asymmetric unit (chains A and B), while highly similar overall, show significant structural divergence in domain 3a. Pro-355, a potential hinge residue [14], is highlighted. The tip of loop 3a was not visible in the Vps16_CTD_–Vps33 complex. (D) Superposition with open and closed Munc18 structures reveals that Vps33 domain 3a adopts an open conformation. Also shown are the relevant regions of open rat Munc18–1 (PDB entry 3PUJ, which includes the N-peptide of syntaxin 4) and closed *M. brevicollis* Munc18 (2XHE, which includes syntaxin 1).

Most of the residues that are conserved among the Vps33-family SM proteins (Fig. 2) map to the hydrophobic core (Fig. 6A). Notably, however, one group of conserved residues forms a surface-exposed cluster (Fig. 6B). This cluster is located near the tip of domain 3a (domain 3a is depicted in ribbon form in Fig. 6C) [16]. Significant conformational variability in this region has been observed in previous SM protein structures [9]–[16]. Notably, the two SM protein structures that contain bound Qa-SNARE four-helix bundles [12], [13], [16] both display closed (or “furled” [14]) conformations for the tip of domain 3a (Fig. 6D). In both of these cases, the closed tip interacts directly with the bound Qa-SNARE. Conversely, the tip of domain 3a adopts open – and rather variable – conformations in the other known SM protein structures [9]–[11], [14], [15]. Within our Vps33-only crystals, the tip of domain 3a adopts two different open conformations dictated by direct, intermolecular tip-tip interactions within each asymmetric unit (Figs. 6C and S2). In the Vsp16_CTD_–Vps33 structure, no interpretable electron density was observed for this region of Vps33 (residues 334–356; Fig. 2), suggesting that in the absence of crystallographic contacts the tip is flexible (Fig. 6C). The exposure of conserved residues caused by the structural plasticity of domain 3a suggests that this region is primed to undergo conformational changes as part of its functional cycle. Finally, it was by modeling the tip of Vps33 domain 3a in a closed conformation that we were able to alleviate the severe clash that was otherwise observed when, as described above, we attempted to model a SNARE complex into the Vps33 binding cleft (Fig. 4C).

## Discussion

The *C. thermophilum* Vps33 and Vps16_CTD_–Vps33 structures provide a first detailed view of an SM protein from the Vps33 family and of its recruitment into a multisubunit tethering complex. Overall, the structure of Vps16_CTD_-bound Vps33 is very similar to the structure of Vps33 alone. In particular, the same positioning of Vps33 domain 1– which represents a striking departure from other SM protein families – is observed in both uncomplexed and complexed Vps33 and may indeed represent an adaptation to promote tighter interaction with Vps16. Importantly, neither the repositioning of domain 1 nor the bound Vps16_CTD_ would obviously preclude the binding of a SNARE complex in the presumptive SNARE-binding cleft (Figs. 1 and 4C).

Previous work on SM proteins has focused significant attention on domain 3a. For example, a random mutagenesis screen for dominant-negative *SEC1* alleles revealed a clustering of mutations that inhibit growth in domain 3a [64], while an extensive mutagenic study to determine the role of Sec1 before and after vesicle docking revealed domain 3a mutants with defects in SNARE complex assembly and binding to pre-formed SNARE complexes [7]. In addition, a *S. cerevisiae* temperature-sensitive allele (E363G) that maps to the tip of domain 3a (Glu-346 in *C. thermophilum* Vps33) inhibits an in vitro fusion assay at a stage after docking but before content mixing [60]. These studies highlight the importance of domain 3a in SNARE complex assembly and imply a function in multiple steps of the fusion reaction. The structures of Vps33 presented here reinforce the idea that flexibility in this conserved region is a shared property among SM proteins and is likely a prerequisite for functional interaction with assembling and/or assembled SNARE complexes. Further biochemical and structural analysis, especially with Vps33 in the context of the HOPS and/or CORVET complexes, will be needed to determine the exact role of domain 3a in SNARE assembly and function in vivo.

While the central feature of SM proteins is their interaction with SNARE proteins, the SNARE interaction profile for each SM protein family is remarkably divergent [1], [3], [4]. As noted by Lobingier and Merz [22], SM proteins fall into two broad classes: class I proteins that bind the Qa-SNARE N-peptide (Munc18, Sly1, and Vps45) and class II proteins (Vps33 and Sec1) that do not. Our Vps33 structure, the first of a class II SM protein, makes it clear why Vps33 is unable to bind N-peptides. Whereas class I structures feature a binding groove with two conserved pockets – one to accommodate an Arg side chain and one to accommodate a hydrophobic side chain [11]–[15] – the class II Vps33 structure reveals that both binding pockets are filled by bulky residues. Given the N-peptide’s apparent role in localizing class I SM proteins to SNARE complexes, an alternative strategy would be needed for class II SM proteins. In the case of Vps33, it may fall upon other subunits within the HOPS/CORVET complexes – by interacting with SNAREs, Rabs, and/or membrane lipids – to recruit the SM protein to the site of SNARE action. Another class II SM protein, Sec1, may likewise depend on other factors, such as the exocyst complex, for recruitment [65].

While SM proteins have apparently diverged with regard to their interactions with individual, uncomplexed SNAREs, most if not all SM proteins seem to share an ability to bind to the four-helix bundles formed by assembled SNARE complexes [18]–[27]. No structure of an SM protein bound to a SNARE complex has thus far been reported. Nonetheless, it is widely assumed – based largely on Munc18–syntaxin 1 structures [12], [13], [16] – that the most likely site for SNARE bundle binding is the central cleft between domains 1 and 3a (but see [7], [66]). By binding to the four-helix bundles of assembled SNARE complexes, SM proteins may help catalyze membrane fusion reactions [1], [4], [18]. Reconstitution experiments have demonstrated that the HOPS complex, presumably through the action of its Vps33 subunit, prevents the disassembly of correctly paired, membrane-bridging trans-SNARE complexes by Sec18/NSF [67]. Conversely, pre-incubation of HOPS with soluble SNARE complexes inhibits the fusion reaction [21]. These and other findings lead to the hypothesis that a primary function of Vps33 is to bind to and prevent disassembly of trans-SNARE complexes [21], [22], [67].

Much work remains in developing a more comprehensive understanding of HOPS/CORVET structure and function. The intact HOPS and CORVET complexes each contain, besides Vps16 and Vps33, four additional subunits. The detailed characterization of their assembly and interaction with functional partners, including SNAREs and Rab proteins, stands as a fundamental but challenging goal for future efforts.

## Supporting Information

Figure S1 **Vps16_CTD_ structural homology.** Proteins with structural homology to Vps16_CTD_ as identified by Dali [63] are shown, after superimposition onto Vps16_CTD_, in two orthogonal views. Vps16_CTD_ helices are represented as yellow cylinders, and those in the structural homologs are in cyan. Nup120 (Dali Z score = 8.0) and coatomer α subunit (Z = 6.3) overlay helices α1–10 of Vps16_CTD_, while the clathrin heavy chain (Z = 6.9) overlays helices α13–17 of Vps16_CTD_.
(TIF)

Figure S2 **Vps33 monomer crystal packing.** (A) The two independent copies of Vps33 are shown in cartoon representation. No significant contacts exist between chain A and chain B except for the distal tips of domains 3a. (B) A small portion of domain 3a from each monomer (residues 319–380) is highlighted to demonstrate the contact surface between monomers in the unit cell. This region is the only area of significant structural deviation between the two copies of Vps33 and is undoubtedly influenced by crystal contacts.
(TIF)
