# Supplementary figures and images for: Crystal Structures of the Sec1/Munc18 (SM) Protein Vps33, Alone and Bound to the Homotypic Fusion and Vacuolar Protein Sorting (HOPS) Subunit Vps16*
Source: PLoS One. 2013 Jun 26;8(6):e67409. doi: 10.1371/journal.pone.0067409 (PMC3693963; doi:10.1371/journal.pone.0067409)

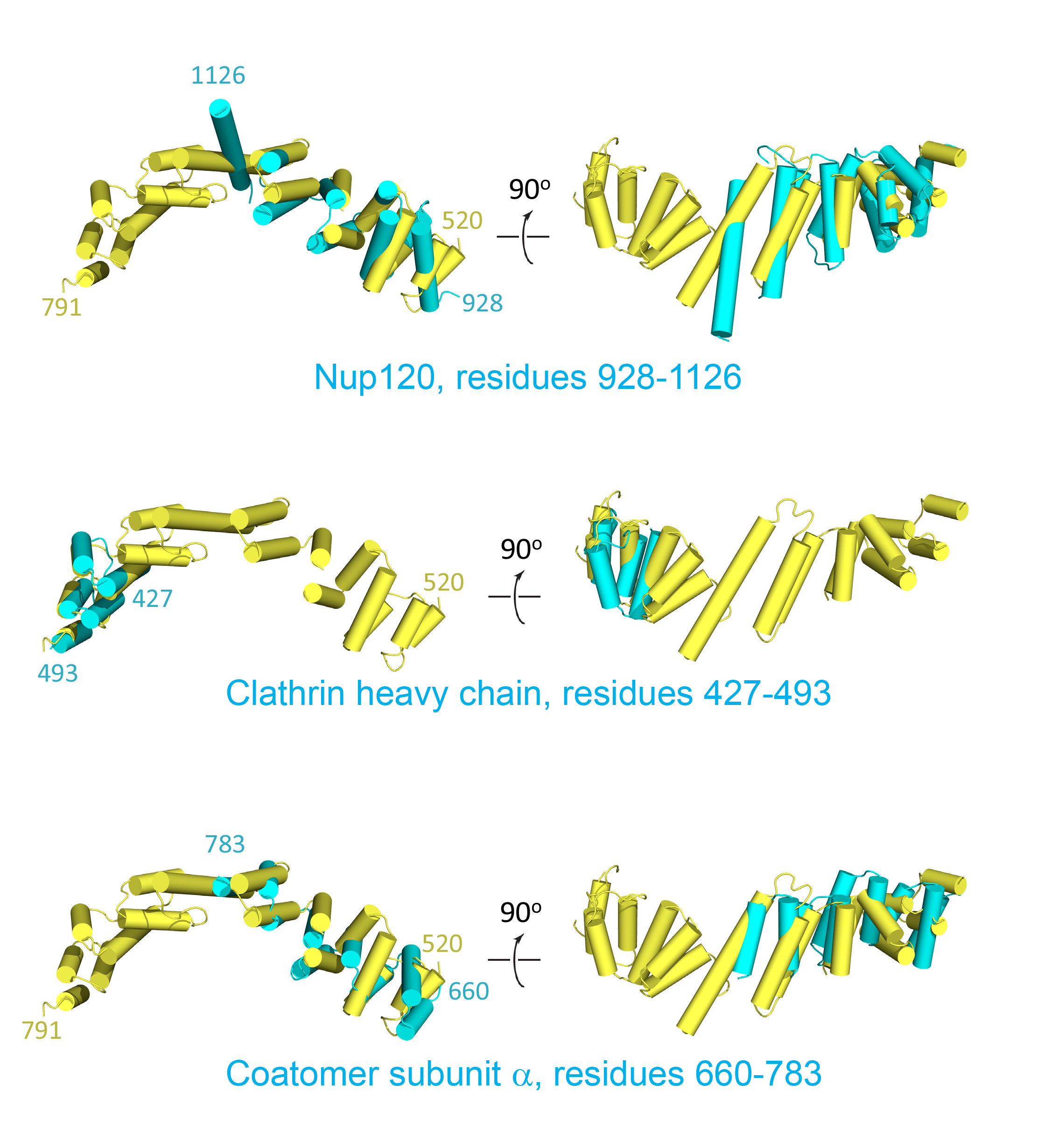

Supplement: Figure S1 — Vps16CTD structural homology. Proteins with structural homology to Vps16CTD as identified by Dali [63] are shown, after superimposition onto Vps16CTD, in two orthogonal views. Vps16CTD helices are represented as yellow cylinders, and those in the structural homologs are in cyan. Nup120 (Dali Z score = 8.0) and coatomer α subunit (Z = 6.3) overlay helices α1–10 of Vps16CTD, while the clathrin heavy chain (Z = 6.9) overlays helices α13–17 of Vps16CTD. (TIF) [file pone.0067409.s001.tif]

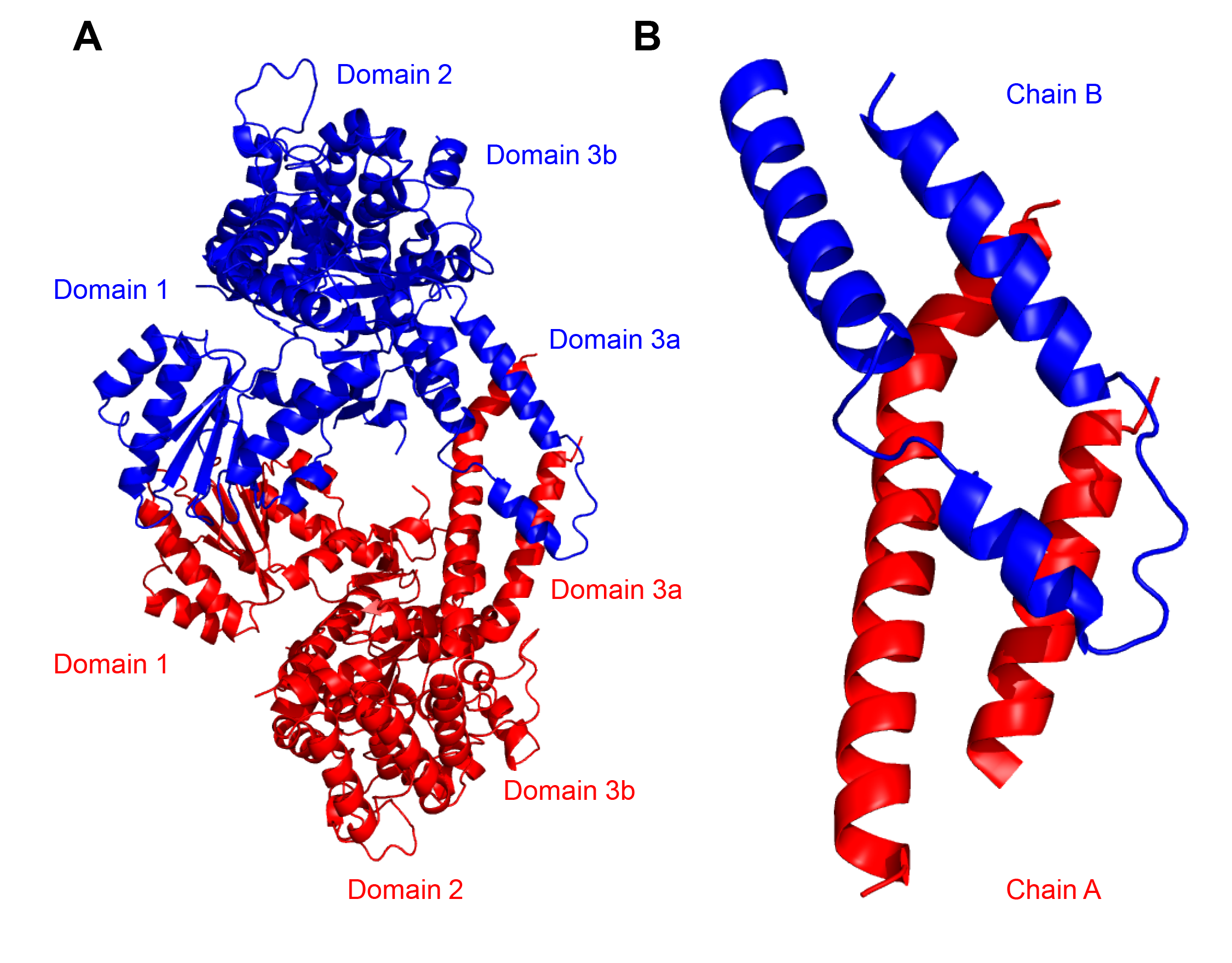

Supplement: Figure S2 — Vps33 monomer crystal packing. (A) The two independent copies of Vps33 are shown in cartoon representation. No significant contacts exist between chain A and chain B except for the distal tips of domains 3a. (B) A small portion of domain 3a from each monomer (residues 319–380) is highlighted to demonstrate the contact surface between monomers in the unit cell. This region is the only area of significant structural deviation between the two copies of Vps33 and is undoubtedly influenced by crystal contacts. (TIF) [file pone.0067409.s002.tif]
